# Comparative Analysis of Prognostic Scores for Predicting Mortality and the Need for Mechanical Ventilation in Patients With Acute Exacerbation of Chronic Obstructive Pulmonary Disease Presenting to the Emergency Department

**DOI:** 10.7759/cureus.82374

**Published:** 2025-04-16

**Authors:** Siddharth Singh, Anand Dev, Abhay Kumar, Santosh Kumar, Shivani Sinha, Santosh Kumar Nayan

**Affiliations:** 1 Emergency Medicine, Indira Gandhi Institute of Medical Sciences, Patna, IND; 2 Critical Care Medicine, Indira Gandhi Institute of Medical Sciences, Patna, IND; 3 General Medicine, Indira Gandhi Institute of Medical Sciences, Patna, IND; 4 Community Medicine, Indira Gandhi Institute of Medical Sciences, Patna, IND

**Keywords:** aecopd, bap65, decaf score, hospital mortality, mechanical ventilation

## Abstract

Objective

This study aimed to evaluate and compare the predictive accuracy of five established clinical scoring systems - CURB65, BAP65, qSOFA, DECAF, and NEWS - in forecasting in-hospital mortality and the need for mechanical ventilation among patients presenting with acute exacerbation of chronic obstructive pulmonary disease (AECOPD) in the emergency department.

Methods

An observational, cross-sectional study was conducted over a 12-month period (January to December 2023) in the emergency department of Indira Gandhi Institute of Medical Sciences, Patna. A total of 200 patients aged 18 years and older with AECOPD were enrolled. Clinical, laboratory, radiographic, and electrocardiographic data were collected to calculate the prognostic scores. The primary outcome was in-hospital mortality; the secondary outcome was the requirement for mechanical ventilation. Statistical analyses were performed using IBM SPSS Statistics for Windows, Version 26.0 (Released 2019; IBM Corp., Armonk, NY, USA) and included logistic regression, receiver operating characteristic curve analysis, calibration assessment, and evaluation of seasonal variations in score performance.

Results

Among the five scoring systems, the DECAF score showed the highest predictive accuracy with an area under the curve (AUC) of 0.80, followed by BAP65 (0.75), NEWS (0.72), CURB65 (0.70), and qSOFA (0.65). DECAF had the strongest association with hospital mortality (OR: 2.8, 95% CI: 1.7-4.5) and need for mechanical ventilation (OR: 2.3, 95% CI: 1.5-3.6). It also achieved the highest sensitivity (0.85) and specificity (0.75). ANOVA revealed significant differences in AUC values across scores (F = 4.76, p = 0.002). Calibration curves indicated accurate prediction for DECAF and BAP65. Seasonal analysis demonstrated consistent performance for both DECAF and BAP65 throughout the year.

Conclusions

The DECAF score was the most reliable predictor of hospital mortality and mechanical ventilation needs in AECOPD patients presenting to the emergency department. BAP65 also performed consistently well and may serve as a practical alternative, particularly in resource-limited settings. Condition-specific scores like DECAF should be favored over general severity indices for effective risk stratification in COPD.

## Introduction

Chronic obstructive pulmonary disease (COPD) remains a major global public health concern, consistently ranking among the leading causes of morbidity and mortality worldwide. According to WHO, COPD was the third leading cause of death globally in 2019, responsible for approximately 3.23 million deaths - accounting for 6% of all fatalities that year [1]. The burden of COPD is particularly pronounced in low- and middle-income countries, where healthcare infrastructure and resources are often limited [2].

In India, COPD is the second leading cause of death after heart disease. Among adults over the age of 30, the estimated prevalence is 4.2% in urban areas and 6.5% in rural regions [3]. Major risk factors contributing to this high disease burden include tobacco smoking, exposure to air pollution, and occupational hazards.

Acute exacerbation of COPD (AECOPD) - marked by the sudden worsening of respiratory symptoms - is a key driver of increased healthcare utilization, frequently leading to hospitalizations and readmissions. These episodes accelerate disease progression, reduce quality of life, and increase mortality risk [4]. In addition to clinical symptoms, certain laboratory markers, such as the neutrophil-to-lymphocyte ratio, have shown potential for predicting adverse outcomes in AECOPD [5].

To aid in clinical decision-making, several prognostic scoring systems have been developed for AECOPD patients, including DECAF, BAP65, and CURB65 [6,7]. These tools help estimate the risk of hospital mortality and the need for intensive interventions, enabling clinicians to make more informed and timely treatment decisions. Comparing the performance of these scoring systems is essential to determine which tools offer the most reliable prognostic value. Identifying the most effective systems can lead to better allocation of healthcare resources and improved patient outcomes.

This study aimed to assess and compare the predictive accuracy of five established clinical scoring systems - CURB65, BAP65, qSOFA, DECAF, and NEWS - in forecasting hospital mortality and the need for mechanical ventilation in patients presenting to the emergency department with AECOPD. The primary objective was to evaluate each scoring system’s sensitivity, specificity, and overall discriminative ability in stratifying patients based on their risk of severe outcomes.

Secondary objectives included analyzing the individual components of these scoring systems to identify which parameters were most strongly associated with mortality and the requirement for mechanical ventilation. The study also assessed the predictive stability of each tool over a six-month period, considering seasonal variations, and aimed to provide comparative insights to support clinical decision-making in resource-limited settings.

## Materials and methods

This observational, cross-sectional study was conducted over a 12-month period, from January to December 2023, in the emergency department of Indira Gandhi Institute of Medical Sciences (IGIMS), Patna - a tertiary care teaching hospital offering specialized services for respiratory diseases. A consecutive non-probability sampling technique was used to enroll all eligible adult patients presenting with AECOPD during the study period. Ethical approval was obtained from the Institutional Ethics Committee of IGIMS, and written informed consent was secured from all participants prior to enrollment.

A total of 200 adult patients (aged 18 years and above) with AECOPD were prospectively enrolled. Patients were excluded if they had acute conditions unrelated to the respiratory system, were referred from other institutions, or had incomplete clinical records. Data were collected at the point of care using a structured proforma, which captured demographic details, clinical presentation, laboratory values, radiographic findings, and electrocardiographic parameters necessary for calculating five prognostic scores.

The scoring systems evaluated in this study were CURB65, BAP65, qSOFA, DECAF, and NEWS. CURB65 assessed confusion, blood urea levels, respiratory rate, blood pressure, and age ≥65 years. BAP65 included blood urea nitrogen, altered mental status, pulse rate greater than 109 beats per minute, and age ≥65 years. The qSOFA score considered respiratory rate ≥22 per minute, altered mental status, and systolic blood pressure ≤100 mmHg. The DECAF score comprised dyspnea grade, eosinopenia, radiological consolidation, acidemia, and the presence of atrial fibrillation. NEWS was calculated based on six physiological parameters: respiratory rate, oxygen saturation, systolic blood pressure, pulse rate, temperature, and level of consciousness. All five scores were calculated at the time of presentation to the emergency department, using information gathered from the initial clinical assessment, laboratory investigations, chest radiography, and ECG findings at admission.

The primary outcome was in-hospital mortality. The secondary outcome was the requirement for mechanical ventilation during the emergency department visit or the subsequent hospital stay.

Statistical analysis

Descriptive statistics, including means, SDs, and frequency distributions, were used to summarize patient demographics and outcome variables. Logistic regression analysis was employed to evaluate the association between each scoring system and the primary outcomes, with adjustments made for age, comorbidities, and prior exacerbation history. The discriminative ability of each scoring system was assessed using receiver operating characteristic (ROC) curve analysis, and the area under the curve (AUC) was reported.

Comparative analyses were carried out using chi-square tests for categorical variables and ANOVA for continuous variables. Chi-square values, F-statistics, and corresponding p-values were reported for each comparison. To further assess diagnostic performance, sensitivity, specificity, positive predictive value, and negative predictive value were calculated for each scoring system.

All statistical analyses were performed using IBM SPSS Statistics for Windows, Version 26.0 (Released 2019; IBM Corp., Armonk, NY, USA), and a p-value of <0.05 was considered statistically significant.

## Results

Among the 200 patients enrolled in the study, the mean age was 65.3 ± 12.4 years, and 65% (n = 130) were male. Half of the patients (50%, n = 100) were current smokers, while 30% (n = 60) had a history of smoking but had since quit. Hypertension was the most common comorbidity, present in 60% (n = 120) of patients, followed by cardiovascular diseases in 45% (n = 90) and diabetes mellitus in 40% (n = 80). In terms of exacerbation history, 45% (n = 90) of patients had experienced two to three exacerbations in the past year, while 15% (n = 30) had more than three. Baseline demographic and clinical characteristics are detailed in Table 1.

**Table 1:** Baseline characteristics of the population under study (n = 200)

Characteristic	n	%
Age (mean ± SD)	-	65.3 ± 12.4 years
Gender		
Male	130	65%
Female	70	35%
Smoking status		
Current smoker	100	50%
Former smoker	60	30%
Never smoker	40	20%
Comorbidities		
Hypertension	120	60%
Diabetes mellitus	80	40%
Cardiovascular diseases	90	45%
Previous exacerbations		
0-1	80	40%
2-3	90	45%
>3	30	15%

Distribution of scores among study participants

Table 2 presents the distribution of prognostic scores among the study participants. Each scoring system - CURB65, BAP65, qSOFA, DECAF, and NEWS - is categorized to show the frequency and percentage of patients falling within each score range. Scores exceeding 3 were grouped together, as they reflected overlapping abnormal clinical parameters. This distribution highlights how each scoring tool stratified the risk levels among AECOPD patients upon presentation to the emergency department.

**Table 2:** Distribution of scores among study participants (n = 200) This table presents the distribution of scores across five prognostic scoring systems — CURB65, BAP65, qSOFA, DECAF, and NEWS — among the 200 study participants. Each row corresponds to a specific score category, indicating the number and percentage of patients classified within that category.

Score category	CURB65	Percentage (%)	BAP65	Percentage (%)	qSOFA	Percentage (%)	DECAF	Percentage (%)	NEWS	Percentage (%)
0	30	15%	40	20%	50	25%	20	10%	10	5%
1	50	25%	60	30%	60	30%	40	20%	30	15%
2	60	30%	50	25%	40	20%	60	30%	50	25%
3	40	20%	30	15%	30	15%	40	20%	60	30%
4	20	10%	20	10%	15	7.50%	30	15%	40	20%
5					5	2.50%	10	5%		

Outcome measures

Table 3 summarizes the outcome measures related to hospital mortality and the need for mechanical ventilation among the study participants. Of the 200 patients, 40 (20%) died during hospitalization, while 60 (30%) required mechanical ventilation either during their emergency department visit or subsequent hospital stay. These outcomes served as the foundation for assessing the predictive performance of the five scoring systems.

**Table 3:** Outcome measures (n = 200) This table summarizes the primary clinical outcomes observed in the study population (n = 200), detailing the number and percentage of patients who experienced hospital mortality or required mechanical ventilation during their emergency department visit or subsequent hospitalization.

Outcome	Frequency (n = 200)	Percentage (%)
Hospital mortality	40	20%
Mechanical ventilation	60	30%

Predictive performance of scoring systems

To evaluate the association between each clinical scoring system and the primary outcomes - hospital mortality and the need for mechanical ventilation - logistic regression analysis was conducted. The analysis revealed that both outcomes increased significantly with scores ≥2, aligning with findings from previous qSOFA validation studies. Table 4 presents the results, including ORs and their corresponding 95% CIs, offering a quantitative assessment of how effectively each scoring system predicts adverse outcomes in AECOPD patients.

**Table 4:** Logistic regression analysis This table presents the results of logistic regression analysis evaluating the relationship between each clinical scoring system and two key outcomes: hospital mortality and the need for mechanical ventilation. ORs greater than 1 indicate an increased risk associated with higher scores. The 95% CIs represent the range within which the true OR is expected to fall. Statistically significant associations are indicated when the CI does not include 1, with p-values <0.05 considered statistically significant.

Scoring system	Hospital mortality OR (95% CI)	p-Value	Mechanical ventilation OR (95% CI)	p-Value
CURB65	2.1 (1.3-3.4)	0.002	1.8 (1.2-2.9)	0.006
BAP65	2.4 (1.5-3.8)	0.001	2.0 (1.3-3.1)	0.004
qSOFA	1.9 (1.2-3.0)	0.008	1.7 (1.1-2.8)	0.012
DECAF	2.8 (1.7-4.5)	<0.001	2.3 (1.5-3.6)	<0.001
NEWS	2.2 (1.4-3.5)	0.002	2.1 (1.4-3.3)	0.003

Table 4 shows that the DECAF score exhibited the strongest association with both hospital mortality (OR: 2.8, 95% CI: 1.7-4.5) and the need for mechanical ventilation (OR: 2.3, 95% CI: 1.5-3.6), followed closely by BAP65, which also demonstrated significant predictive performance (OR: 2.4 for mortality and 2.0 for mechanical ventilation). The qSOFA and CURB65 scores showed a modest association with outcomes, while NEWS displayed intermediate predictive capacity.

These findings suggest that DECAF is the most reliable scoring system among those assessed for identifying patients at higher risk of mortality and the need for ventilatory support in the emergency department setting.

ROC curve analysis

Following the logistic regression analysis, we performed ROC curve analysis to further assess and compare the discriminative performance of each prognostic scoring system. While logistic regression quantifies the strength of association between each scoring system and clinical outcomes, ROC curve analysis evaluates the model's overall ability to distinguish between patients who did and did not experience the outcomes - specifically, hospital mortality and the need for mechanical ventilation.

The ROC curve plots the true positive rate (sensitivity) against the false positive rate (1-specificity) at various threshold levels, providing both a visual and quantitative measure of each model’s diagnostic accuracy. The AUC serves as a summary metric reflecting the discriminatory power of each scoring tool, with values closer to 1.0 indicating higher accuracy.

**Figure 1:**
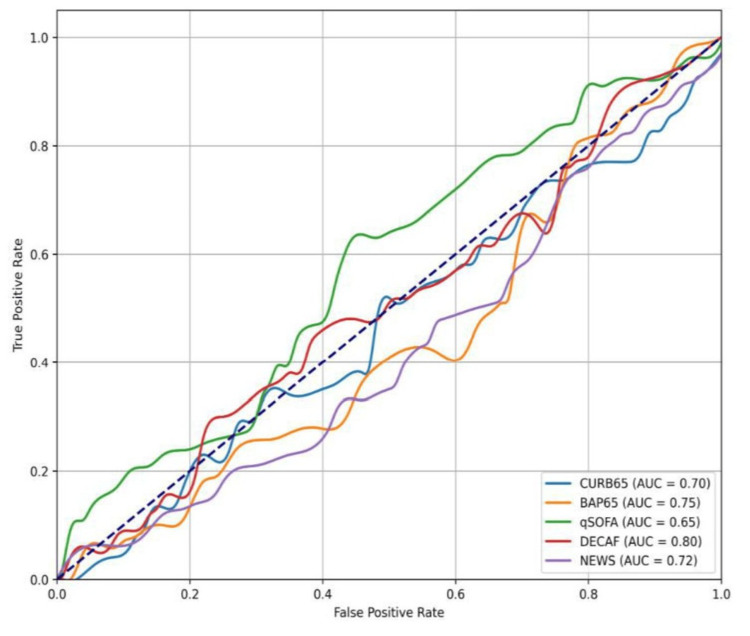
ROC curves for each scoring system This figure compares the ROC curves of five prognostic scoring systems — CURB65, BAP65, qSOFA, DECAF, and NEWS — in predicting hospital mortality and the need for mechanical ventilation in AECOPD patients. The AUC values reflect the discriminative ability of each system, with DECAF demonstrating the highest AUC (0.80), followed by BAP65 (0.75), NEWS (0.72), CURB65 (0.70), and qSOFA (0.65). These results suggest that DECAF is the most accurate tool among those evaluated. AECOPD, acute exacerbation of chronic obstructive pulmonary disease; AUC, area under the curve; ROC, receiver operating characteristic

Figure 1 displays the ROC curves and AUC values for the five scoring systems (CURB65, BAP65, qSOFA, DECAF, and NEWS), providing a comparative visualization of their performance in identifying high-risk AECOPD patients who require critical intervention.

As illustrated in Figure 1, the DECAF score demonstrated the highest discriminative ability for predicting both hospital mortality and the need for mechanical ventilation, with an AUC of 0.80. This was followed by BAP65 (AUC = 0.75), NEWS (AUC = 0.72), and CURB65 (AUC = 0.70), all of which showed moderate predictive accuracy. The qSOFA score had the lowest AUC (0.65), indicating limited discriminative performance compared to the other scoring systems. These findings emphasize the superior prognostic value of disease-specific tools like DECAF in the emergency management of AECOPD.

Statistical comparison of discriminative performance

To further substantiate the visual differences observed in the ROC curves, we performed an ANOVA to statistically compare the AUC values of the five scoring systems. This analysis helped determine whether the observed variations in predictive performance were statistically significant across the scoring systems. The mean AUC, F-statistic, and corresponding p-values for each score are presented in Table 5. These results confirm the superior discriminative ability of the DECAF and BAP65 scores, with statistically significant differences in performance compared to the other tools.

**Table 5:** Comparative AUC values and ANOVA results for predictive performance of scoring systems This table presents the comparative AUC values derived from ROC analysis for each scoring system, along with the F-statistics and p-values obtained from ANOVA to assess the statistical significance of differences in predictive performance. A higher AUC indicates better discriminative ability, while p-values <0.05 indicate statistically significant differences between the scoring systems. AUC, area under the curve; ROC, receiver operating characteristic

Scoring system	AUC	F-statistic	p-Value (ANOVA)
CURB65	0.7	6.21	0.0003
BAP65	0.75	10.85	<0.001
qSOFA	0.65	3.12	0.028
DECAF	0.8	14.92	<0.001
NEWS	0.72	8.34	0.0012

Sensitivity and specificity analysis

**Figure 2:**
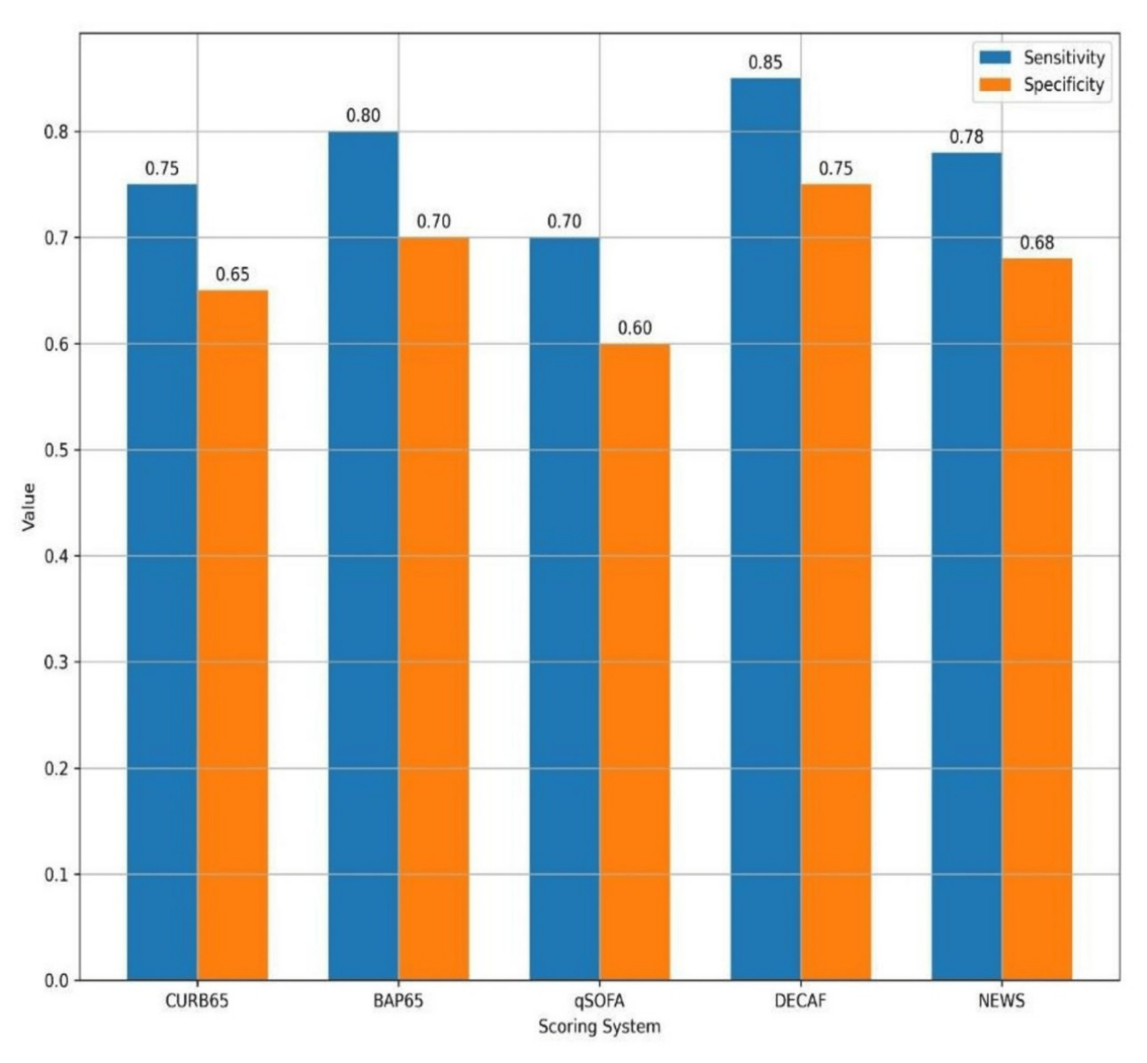
Comparison of sensitivity and specificity across different scoring systems The bar chart illustrates the sensitivity and specificity of each prognostic scoring system (CURB65, BAP65, qSOFA, DECAF, and NEWS) in predicting hospital mortality and the need for mechanical ventilation in patients with AECOPD. Sensitivity indicates the ability of each score to correctly identify patients who experienced adverse outcomes (true positives), while specificity reflects the ability to correctly identify those who did not (true negatives). Among the scoring systems, DECAF demonstrated the highest sensitivity (0.85) and specificity (0.75), followed by BAP65 with a sensitivity of 0.80 and specificity of 0.70. In contrast, qSOFA exhibited the lowest performance, with a sensitivity of 0.70 and specificity of 0.60. AECOPD, acute exacerbation of chronic obstructive pulmonary disease

To further assess the diagnostic accuracy of the five scoring systems, sensitivity and specificity values were compared, as shown in Figure 2. Sensitivity reflects the ability of each scoring system to correctly identify patients who experienced adverse outcomes (true positives), while specificity measures the ability to accurately identify those who did not experience adverse outcomes (true negatives). These metrics offer practical insights into the balance between identifying high-risk patients and minimizing false positives, which is essential for clinical decision-making in emergency settings.

The DECAF score demonstrated the highest sensitivity (0.85) and specificity (0.75), highlighting its superior ability to accurately identify both high-risk and low-risk AECOPD patients. BAP65 also performed well, with a sensitivity of 0.80 and specificity of 0.70, making it a reliable secondary option. In contrast, qSOFA exhibited the lowest diagnostic performance, with sensitivity at 0.70 and specificity at 0.60, underscoring its limitations in accurately stratifying patient risk in this clinical context.

Component analysis

To better understand the contribution of individual variables within each scoring system, a logistic regression analysis was conducted for hospital mortality and the need for mechanical ventilation. The most predictive components across the five scoring systems are summarized in Table 6, based on their ORs and CIs. This analysis offers valuable insight into the specific clinical parameters that most strongly influence adverse outcomes in AECOPD patients, helping guide clinicians in determining the relative importance of individual score elements.

**Table 6:** Logistic regression analysis of scoring system components for hospital mortality This table summarizes the most predictive individual components from each prognostic scoring system, derived from multivariable logistic regression analysis. The ORs, along with their 95% CIs and p-values, reflect the strength and statistical significance of each component's association with hospital mortality and/or the need for mechanical ventilation. Components with an OR greater than 1 and a p-value less than 0.05 were considered statistically significant predictors.

Component	Scoring system(s)	OR (95% CI)	p-Value
Acidemia	DECAF	2.7 (1.8-4.1)	<0.001
Consolidation	DECAF	2.5 (1.6-3.9)	<0.001
Altered mental status	BAP65, qSOFA	1.8 (1.2-2.7)	0.004
Dyspnea grade	DECAF	2.0 (1.4-3.2)	0.001
Eosinopenia	DECAF	1.8 (1.2-2.8)	0.006
Age ≥65	CURB65, BAP65	1.9 (1.3-2.9)	0.002
Urea/BUN	CURB65, BAP65	2.0 (1.3-3.0)	0.001
Heart rate	NEWS	1.7 (1.2-2.6)	0.005

Temporal stability and seasonal variation

To evaluate the stability of predictive performance across seasonal variations, we assessed the monthly AUC values of each scoring system over the six-month study period. This analysis aimed to determine whether the discriminative power of each tool remains consistent throughout the year or is influenced by temporal factors, such as seasonal infections and environmental changes.

**Figure 3:**
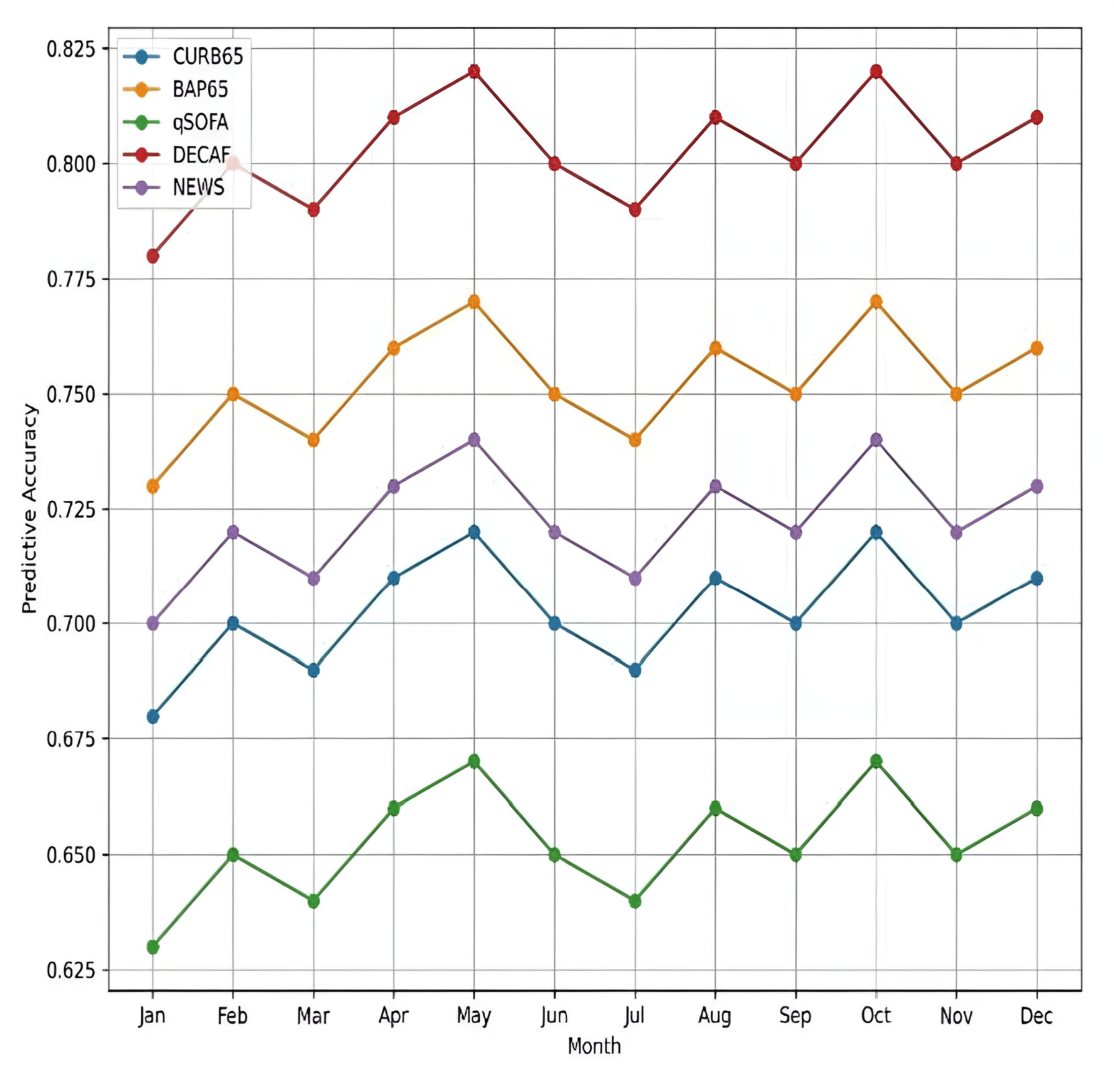
Predictive accuracy of each scoring system over different months Month-wise AUC trends for each scoring system (CURB65, BAP65, qSOFA, DECAF, and NEWS) assessing predictive accuracy for hospital mortality in AECOPD patients. DECAF and BAP65 demonstrated consistent performance across all months, while CURB65 and qSOFA exhibited noticeable variability. AECOPD, acute exacerbation of chronic obstructive pulmonary disease; AUC, area under the curve

As shown in Figure 3, the DECAF score maintained the highest and most stable predictive accuracy throughout the six-month period, with AUC values consistently ranging from 0.78 to 0.82. BAP65 also demonstrated reliable performance, with only minor fluctuations from month to month (AUC 0.73-0.77). In contrast, CURB65 and qSOFA exhibited greater variability, suggesting that seasonal trends, such as respiratory infections and environmental changes, may have influenced their performance. NEWS showed moderate variation. These findings highlight the robustness of DECAF and BAP65 for year-round use in clinical decision-making for AECOPD patients.

Calibration analysis

To further assess the performance and reliability of each scoring system, we conducted a calibration analysis. Calibration evaluates how closely the predicted probabilities of an event (e.g., mortality) align with actual outcomes across different risk levels. Well-calibrated models not only discriminate effectively but also provide accurate risk estimates, which are crucial for clinical decision-making.

**Figure 4:**
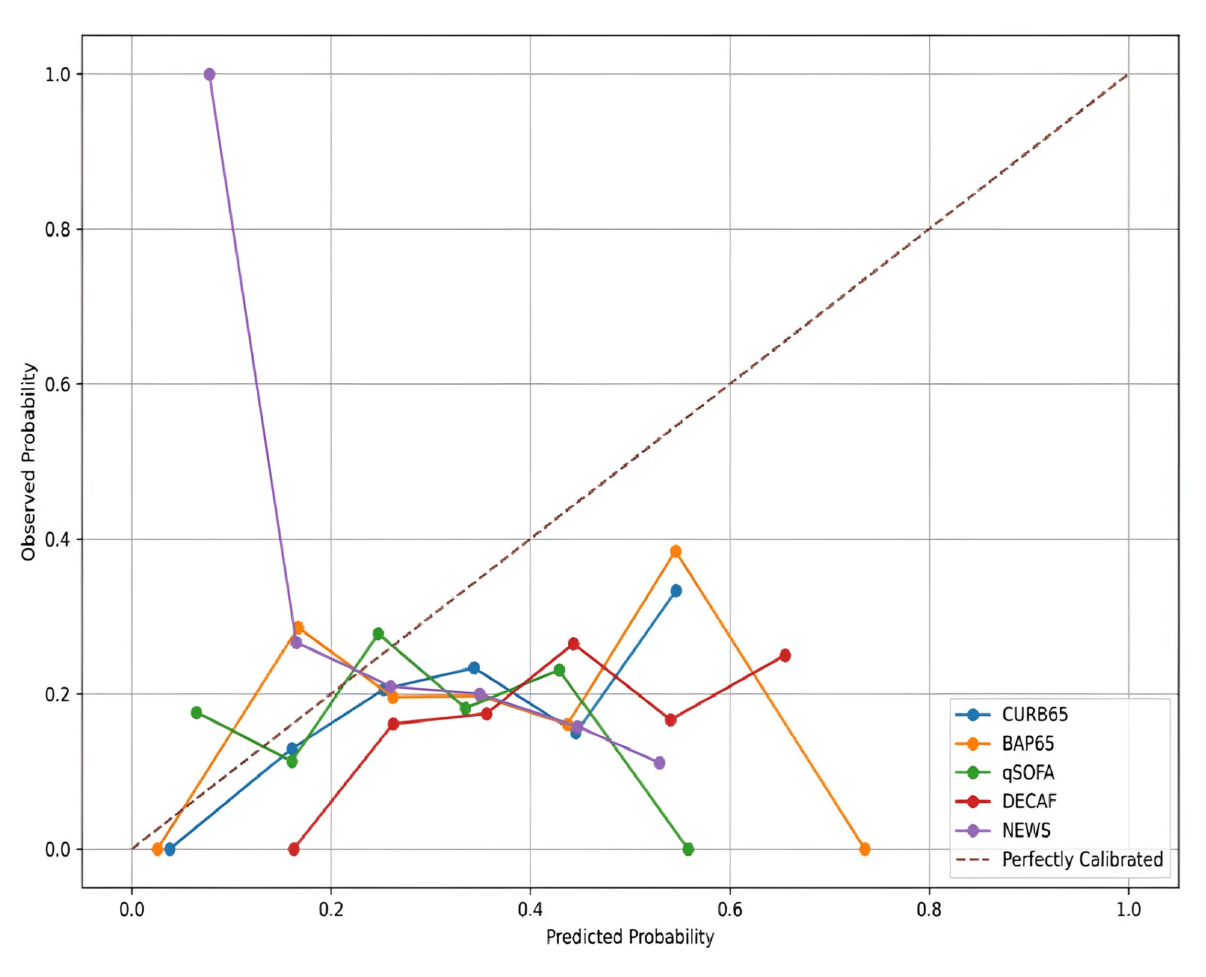
Calibration curves for predictive accuracy of scoring systems in AECOPD patients Calibration curves for the five scoring systems (CURB65, BAP65, qSOFA, DECAF, and NEWS) in predicting hospital mortality among AECOPD patients. The 45-degree reference line represents perfect calibration. DECAF and BAP65 curves closely align with this line, indicating strong agreement between predicted and observed outcomes. CURB65 and NEWS showed modest over- or underestimation at higher risk levels, while qSOFA demonstrated the poorest calibration. AECOPD, acute exacerbation of chronic obstructive pulmonary disease

As depicted in Figure 4, the DECAF and BAP65 scores showed the best calibration, with their curves closely approximating the ideal 45-degree line across all probability thresholds. This indicates a strong agreement between the predicted risks and the actual observed outcomes. CURB65 and NEWS demonstrated reasonable calibration at lower and moderate risk levels but deviated slightly at higher predicted probabilities. In contrast, the qSOFA score showed the least reliable calibration, with significant divergence from the reference line at higher-risk strata, suggesting potential misestimation of mortality risk in sicker patients.

## Discussion

Comprehensive performance of prognostic scoring systems

This study offers a thorough comparison of five widely used prognostic scoring systems - CURB65, BAP65, qSOFA, DECAF, and NEWS - in predicting mortality and the need for mechanical ventilation among AECOPD patients presenting to the emergency department. Our findings reveal that, although all scoring systems exhibited predictive value, the DECAF score consistently showed superior performance across various measures of accuracy and reliability.

Primary findings and comparative performance

The DECAF score demonstrated the strongest predictive capacity for both hospital mortality (OR 2.8, 95% CI: 1.7-4.5) and the need for mechanical ventilation (OR 2.3, 95% CI: 1.5-3.6). This superior performance was further validated by ROC curve analysis, where DECAF achieved the highest AUC (0.80), significantly outperforming the other scoring systems. The high sensitivity (0.85) and specificity (0.75) of DECAF suggest its particular utility in blood urea nitrogen and respiratory rate, which have proven predictive value, as highlighted by Chang et al. (2010) [8].

These findings align with Echevarria et al. (2016) [9], who validated the DECAF score’s predictive utility in both internal and external cohorts, demonstrating its robust performance in predicting in-hospital mortality for COPD exacerbations. However, our results also draw parallels with studies by Chang et al. (2011) [8] and Shorr et al. (2011) [10], which emphasized the utility of CURB65 and BAP65, respectively, for stratifying risk in COPD exacerbations. While DECAF’s disease-specific parameters, such as dyspnea grade and eosinopenia, contribute to its superior performance, other scoring systems also have strengths worth noting. For instance, CURB65 incorporates blood urea nitrogen and respiratory rate, which have proven predictive value, as highlighted by Chang et al. (2010) [8]. Similarly, BAP65’s simplicity and focus on clinically accessible variables make it an effective alternative, as validated by Shorr et al. (2011) [10].

Discriminative ability and pathophysiological insights

ROC and Performance Analysis

The AUC analysis revealed a clear hierarchical pattern in the discriminative performance of the scoring systems. The DECAF score exhibited the highest AUC at 0.80, followed by BAP65 at 0.75, NEWS at 0.72, CURB65 at 0.70, and qSOFA at 0.65. The DECAF score’s superior discriminative ability can be attributed to its comprehensive and disease-specific parameters tailored to COPD exacerbations. For example, eosinopenia has been independently associated with adverse outcomes in AECOPD patients, as supported by Mao et al. [11], who found eosinophil counts below 50/μL to be a strong predictor of 18-month mortality and a marker of more severe infections. Additionally, dyspnea grade, a core component of the DECAF score, directly reflects respiratory compromise - a central pathophysiological feature of AECOPD. The notable AUC difference of 0.15 between DECAF and qSOFA further emphasizes the greater utility of disease-specific scoring systems over generalized tools in predicting poor outcomes in COPD exacerbations.

Pathophysiological Interpretations

The DECAF score’s holistic framework integrates several pathophysiological markers that reflect the multidimensional nature of AECOPD. Dyspnea grade serves as a direct measure of respiratory compromise and is a well-established indicator of disease severity. Eosinopenia, another key parameter, represents a biomarker of systemic inflammatory response and has been increasingly recognized as a predictor of poor outcomes. Consolidation on chest imaging is typically associated with concurrent pneumonia and contributes significantly to the overall disease burden. Acidemia indicates the presence of respiratory failure, marking more severe systemic compromise, while atrial fibrillation reflects underlying cardiovascular instability, which further exacerbates clinical deterioration in AECOPD patients. These components together capture the complex interplay of respiratory, inflammatory, and systemic factors, providing a nuanced and condition-specific risk assessment. Previous studies by Mohanty and Babu (2016) [12] and Xu et al. (2024) [13] have underscored the prognostic value of these elements, particularly eosinopenia, in predicting adverse outcomes in hospitalized patients with COPD exacerbations.

Temporal stability and implementation implications

A particularly valuable finding was the temporal stability of the DECAF score (mean AUC = 0.80, SD = 0.03) across different seasons. This consistency suggests that DECAF could be reliably implemented year-round, an important consideration for institutional protocol development. The observed seasonal variations in the performance of other scoring systems might reflect their sensitivity to factors such as seasonal illness patterns or operational variations in emergency department capacity. These findings align with comparative analyses by Chang et al. (2010) [8] and Acet-Öztürk et al. (2024) [14], which demonstrated the utility of CURB65, BAP65, and DECAF in various clinical contexts. Acet-Öztürk et al. (2024) [14] particularly emphasized the superior predictive power of DECAF and BAP65 for respiratory support needs in hospitalized AECOPD patients.

Seasonal Variations and Their Impact on Predictive Accuracy

The seasonal variation analysis revealed that DECAF and BAP65 maintained stable performance across months, with minimal AUC fluctuations (DECAF: 0.78-0.82; BAP65: 0.73-0.77). In contrast, CURB65 and qSOFA showed significant variability, likely reflecting their vulnerability to external factors such as temperature, humidity, and air quality. These findings highlight the resilience of DECAF and BAP65 in diverse settings while raising questions about the broader applicability of CURB65, NEWS, and qSOFA. Acet-Öztürk et al. (2024) [14] noted that BAP65 and DECAF outperformed other scores in predicting respiratory support needs, reinforcing their reliability for COPD-specific risk stratification. Meanwhile, studies like those by Chang et al. (2010) [8] underscore CURB65’s utility in specific subgroups.

Implications for clinical practice

Risk Stratification

The superior performance of DECAF supports its adoption as the primary scoring tool for AECOPD in emergency department settings. Its ability to predict outcomes with high accuracy enables clinicians to prioritize high-risk patients for intensive care and resource allocation. BAP65, with its relatively simpler parameters, could serve as an alternative when rapid assessments are required.

Limitations of Generalized Systems

qSOFA’s lower accuracy emphasizes the importance of using disease-specific tools for COPD patients, particularly in emergencies where nuanced assessments are critical for guiding clinical interventions.

Seasonal Considerations

The stable year-round performance of DECAF and BAP65 makes them reliable across different seasons. In contrast, CURB65 and qSOFA may require adjustments or supplemental tools during high-variability periods.

Limitations and future directions

Several limitations should be considered when interpreting these results. The single-center nature of the study may limit generalizability to other settings, particularly those with different resource levels or patient populations. Additionally, the study’s observational design means that the impact of scoring system implementation on patient outcomes was not directly assessed.

Future research should focus on enhancing the clinical utility of these findings by prioritizing multicenter validation studies to confirm the consistency of scoring systems like DECAF and BAP65 across diverse healthcare settings. As highlighted by Telukutla et al. (2020) [15], external validation is essential for broader adoption. Including CURB65 and NEWS in such comparative analyses will further clarify their contextual relevance.

Additionally, prospective studies are needed to evaluate how score-guided clinical decisions impact patient outcomes. Exploration of simplified or modified scoring systems could also help retain predictive value while improving feasibility, especially in resource-limited environments.

Finally, cost-effectiveness analyses should be conducted to assess the practicality of implementing these tools at scale, supporting evidence-based resource allocation in emergency care.

## Conclusions

This study provides strong evidence supporting the use of the DECAF score as the preferred prognostic tool for AECOPD patients in the emergency department setting, while also validating BAP65 as a valuable alternative. The findings contribute to the growing body of evidence supporting the use of standardized prognostic scoring systems in acute COPD care and provide practical insights for their implementation in clinical practice.
